# The health-related Millennium Development Goals (MDGs) 2015: Rwanda performance and contributing factors

**DOI:** 10.11604/pamj.2018.31.56.11018

**Published:** 2018-09-26

**Authors:** Médard Nyandekwe, Jean Baptiste Kakoma, Manassé Nzayirambaho

**Affiliations:** 1 University of Rwanda, College of Medicine and Health Sciences, School of Public Health, Kigali, Rwanda

**Keywords:** Rwanda, Millennium Development Goals, Health-related targets

## Abstract

**Introduction:**

The Millennium Development Goals (MDGs) 2015 are the eight international development goals adopted by the Millennium Summit of the United Nations in 2000 to which Rwanda is signatory. In 1990, Rwanda was at least one of the Sub-Saharan Africa countries with poor performance on health-related MDGs indicators. To date, despite the setbacks caused by the 1994 genocide, impressive performance is registered. The objective of the study is to document Rwanda gradual progress to achieving the health-related MDGs 2015 targets from 1990 to 2014/2015.

**Methods:**

The study is retrospective and comparative documenting the period of 1990 to 2014/15.

**Results:**

The performance of Rwanda on health-related MDGs 2015 targets is impressive despite the negative effects of the 1990-1994 civil wars and the 1994 genocide against Tutsi on 1990's levels. In effect, out of 17 health-related MDGs indicators, eleven (11) registered “remarkable” performances, i.e. reached global levels or fastened Vision 2020 targets attainment, two (2) registered “good performances”, i.e. reached basic or revised own targets exhibiting overall impressive performance, while four (4) “weaknesses” are observed, i.e. accused gaps until now. The good governance, Vision 2020 effective implementation, consistent resources invested in health sector and the Rwanda Universal Health Coverage implementation contributed greatly to achieving the above health-related MDGs 2015 performance.

**Conclusion:**

Rwanda performance of health-related MDGS 2015 targets is impressive. However, some relative gaps still persist, and hence should be prioritized while implementing the emerging Sustainable Development Goals.

## Introduction

The Millennium Development Goals (MDGs) are the eight international development goals that were established and adopted by the Millennium Summit of the United Nations in 2000 to which Rwanda is signatory. These goals are the following: Goal 1: eradicate extreme poverty and hunger; Goal 2: achieve universal primary education; Goal 3: promote gender equality and empower women; Goal 4: Reduce child mortality; Goal 5: improve Maternal Health; Goal 6: combat HIV/AIDS, Malaria and other diseases; Goal 7: ensure Environmental Sustainability, and Goal 8: develop a Global Partnership for Development [1]. The first seven goals are mutually reinforcing and are directed at reducing poverty in all its forms. This study addresses only the first, fourth, fifth, sixth, and the seventh health-related goals. The last (8^th^) goal is also concerned by the study as a contributing factor to achieve them. The targets and related indicators are presented in the results section. As regard to the MDGs implementation and Performance, Rwanda displays a unique case. In 1990, Rwanda was one of the Sub-Saharan Africa (SSA) countries with poor performance on health-related MDGs indicators worsened by the civil war from 1990 to 1994 and the 1994 genocide against Tutsi [2, 3]. Whereas most of the publications on Rwandan health-related MDGs focus only on the status aspect without providing a comprehensive and global view of the situation, this study addresses the gap identified by providing following additional information: Persisting gaps up to date; Variation from 1990 up to 2015 including the setbacks resulted from the 1994 genocide against the Tutsi and health gain (from 2000) achieved during the MDGs lifetime; Contributing factors that stimulated and improved performance; and Comparison of current status with WHO African Region and WHO Global Region standards.

The aim of this study is to document Rwanda gradual own progress to achieving the MDGs 2015 health-related targets from 1990 to 2014/2015. Specifically, the study: (1) presents current status of concluded Rwanda health-related MDGs 2015 and thus constituting the baseline for the emerging SDGs; (2) determines variation from the year 1990 to 2014/2015 including the setbacks resulted from the 1994 genocide against Tutsi; (3) highlights persisting gaps up to date; (4) compares current status with international levels and; (5) examines potential contributing factors that stimulated improvement and fastened targets attainment.

## Methods

This is a retrospective and comparative study documenting the period of 1990 to 2015. Periodic national surveys and existing international relevant reports on the topic along with extensive desk review on related data have been conducted. The basic universal official indicative and unanimous goals issued by United Nations (UN) MDGs 2000 and the recent UN 2015 MDGs Report [4] have been utilized with regards to MDGs goals, targets and indicators to measure the progress and compare the Rwanda status with worldwide status. Rwanda own tracked sub-indicators were excluded if no comparative data was retrieved in recent reviewed WHO and UN MDGs reports. As all Rwanda health-related MDGs indicators were dramatically reversed during the horrors of early 1990s, some of them were referred to 2000 levels as starting line in the attempt of setting realistic basic indicators and targets. Along the MDGs 2015 lifetime, some Rwanda basic targets were revised by the Government of Rwanda (GOR) in order to adapt them to the Vision 2020 [5] expectations. Revisions were done according to observed and evidence-based improvements in the Rwanda epidemiological profile, the Rwanda macro-economy, and the Rwanda social health sector. These revisions were found in the Economic Development and Poverty Reduction Strategy 2008-2012 (EDPRS1) [6]. Latest revised targets set for 2015 have been supplied by the Result Framework on Third Health Sector Strategic Plan 2012-2018 [7] and by the MDGs Rwanda Progress Report 2013 [8].

## Results

**Figure 1:**
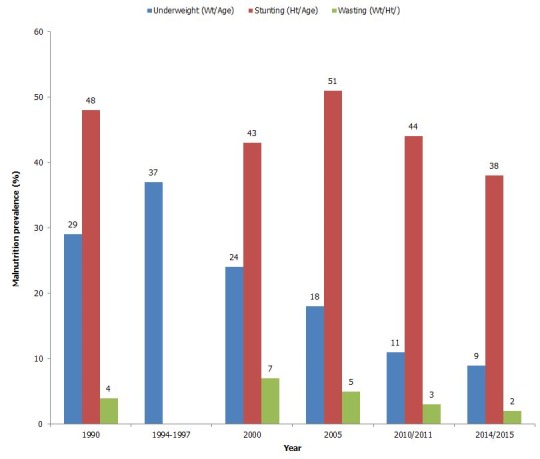
Evolution of the nutritional health status from 1990 to 2014/2015

**Figure 2:**
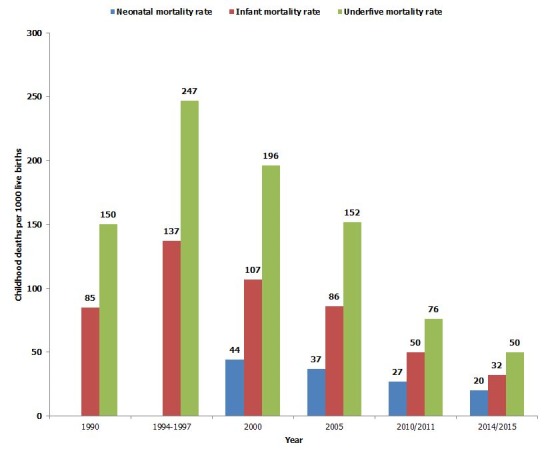
Trend in childhood mortality (deaths per 1000 live births) from 1990 to 2014/2015

**Figure 3:**
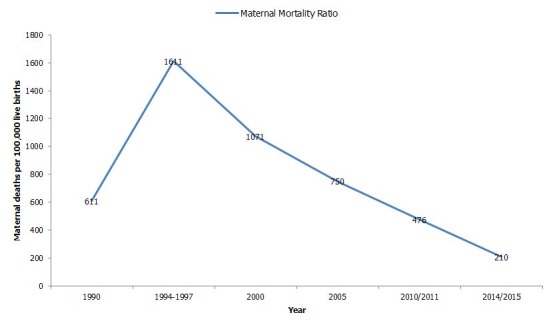
Trend in maternal mortality ratio (deaths per 100,000 live births) from 1990 to 2014/2015

**Figure 4:**
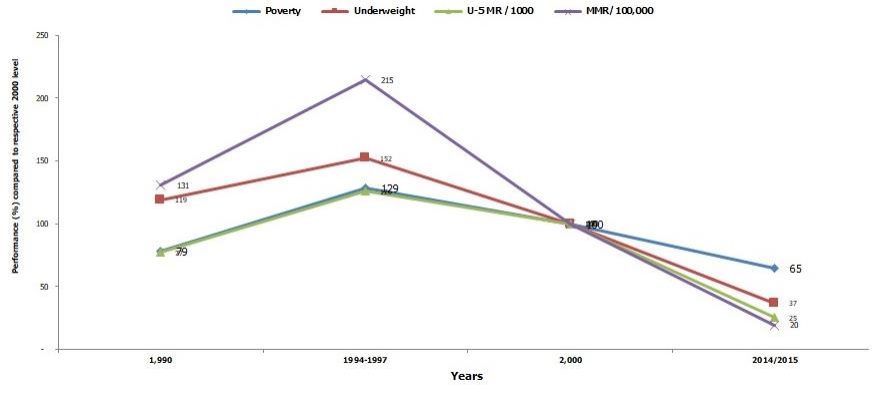
Trend of main indicators’ performance from 1990 to 2014/2015 compared to respective 2000 level

The results are framed around the (i) Gradual progress from the starting line (1990) to 2015 comprised in main results' section and in Figure 1, Figure 2, Figure 3; (ii) Performance of main Rwanda MDGs indicators between 1990 and 2014/2015 illustrated by Figure 4; (iii) Persisting gaps up to date. If any, the gap is presented in simple deviation (Actual-Target) from the target and not in relative ((Actual-Target)/Target)*100 to avoid any confusion. As advantage, the gap presented using “Actual-Target” will keep the same nature of indicator defined in absolute number instead of transforming all gaps in percentages. (iv) Contributing factors that stimulated and improved performance.

***GOAL 1: eradicate extreme poverty and hunger***

***Target 1: Between 1990 and 2015, halve the proportion of people living below the poverty line***

**Indicator 1: proportion of population living below the poverty line**

Globally, the poverty line indicator was defined by “the proportion of the population living on less than $1 a day” [1] and presently less than $1.25 a day [4]. According to the first Integrated Household Living Conditions Survey (EICV1 2000/2001) [9], the Rwandan national poverty line defined in 2001 was calculated at RWF 64,000 and the extreme poverty line was calculated at RWF 45,000 constant prices 2001 per annum per adult equivalent. The poverty line indicator was 47.5% in 1990 [10], 77.8% in 1994 [10], 60.4% in 2000/2001 [9], 44.9% in 2010 [11] and dropped to 39.1% in 2013/2014 [12]. To be realistic, Rwanda decided to set the target at halving the 2000/2001's poverty line i.e. 30.2% by 2015, not achieved, instead of halving the 1990s' level i.e. 23.8%. In the same period, the extreme poverty rate in Rwanda fell from 41.3% in 2000/2001 [9] to 16.3% in 2013/2014 [12]; the Rwanda own basic target set i.e. the half of 2000/2001 level (20.7%) was achieved.

***Target 2: Between 1990 and 2015, halve the proportion of people who suffer from hunger***

**Indicator 4: prevalence of underweight children under-five years of age**

**Weight-for-age (underweight):** The 1992's level of that form of malnutrition was 29% [13], 37% between 1994-1997, 24.3% in 2000, 18% in 2005, 11% in 2010/2011 whilst the recent status of the indicator was 9% [14], revealing the achievement of the basic target set at 14.5% by 2015.

**Height-for-age (stunted):** The 1992's level was 48% [13], 43% in 2000, rose to 51% in 2000, declined to 44% in in 2010/2011, and thirty-eight percent (38%) of children were stunted [14], according to RDHSV 2014/2015; the basic target is not achieved and the gap is 14% of reduction compared to basic target set at 24% and revised at 24.5% [8].

**Weight-for-height (wasted):** In 1990, in Rwanda, the percentage of under-five children wasted was 4% [13], rose to 7% in 2000 decreased to 5% in 2005, 3% in 2010/2011 and to 2% in 2014/2015 [14]. The related Rwandan target set at 2% by 2015 was then achieved. The progress achieved by Rwanda in reducing malnutrition is illustrated by the Figure 1.

***GOAL 4: reduce child mortality***

***Target 5: Between 1990 and 2015, reduce the under-five mortality rate by two-thirds***

**Indicator 13: under-five mortality rate per 1000 live births**

In 1990, the indicator was 151.8 deaths/1000 [2], rose to 247/1000 in 1994 [2], decreased to 196.2 in 2000, 152% in 2005, 76% in 2010/2011 and to 50 deaths /1,000 in 2014/2015 [14] as illustrated by Figure 2, attaining exactly the basic target set at 50 /1000 by 2015. The gap worth 20 deaths to be reduced persists compared to the Rwanda revised target set at 30 deaths per 1000 live births [7].

**Indicator 14: infant mortality rate per 1000 live births**

From 85/1000 in 1990 [2] , the indicator increased to 137 /1000 in 1994 [2] and declined to 107% in 2000, 86% in 2005, 50% in 2010/2011 up to 32/1,000 in 2014/2015 [14] as indicated by Figure 2. Neither the basic target set at 28/1,000 nor the revised target set at 25/1,000 [7] were achieved. The persisting gap is 7 death /1,000 of reduction. Rwanda tracked also neonatal mortality rate per 1000 since 2000; it recorded 44% in 2000, 37% in 2005, 27% in 2010/2011 and 20% in 2014/2015 [14].

**Indicator 15: proportion of one-year-olds children immunized against measles**

The indicator dropped significantly from 83% in 1990 [13] to 42% in 1994-1995 [10] and rose gradually up to 95.2% in 2014/2015 [14], achieving the revised target set at 95%.

**Indicator 16: Maternal Mortality Ratio (MMR) per 100,000 live births**

The indicator was estimated at 1400/100,000 in 1990 [13], jumped to 2300/100,000 in 1995 [2] and declined to 1071 in 2000, 750 in 2005, 476 in 2010/2011 up to 210/100,000 in 2014/2015 [14] versus 268/100,000 as initial own target based on 2000 level worth 1071/100,000 as illustrated by the Figure 3. The target is then surpassed.

**Indicator 17: proportion of births attended by skilled health personnel**

The indicator decreased from 25% in 1990 (RDHSI) [13] to 19% in 1996 [2], gradually increased to 35%, 39 %, 69% and 90.7% in years 2000, 2005, 2010, and 2014/2015[7]; the basic target set at 75% by 2015 is surpassed.

***Target 7: By 2015, to have halted and begun to reverse the spread of HIV/AIDS***

**Prevalence of HIV prevalence amongst men and women aged 15-49 years**

A comprehensive survey conducted in five HIV sero-surveillance sentinel sites revealed a HIV prevalence rate of 11.2% in 2000 [**15**]. The systematic survey conducted national wide in 2003 revealed a HIV prevalence worth 5.1% among general population aged 15-49 years [16] and two years later at 3% [17]. Since then to present, the HIV prevalence remains the same (3%) [14]. It should be naïve to confirm that the basic target set at 5% by 2020 [5] was attained within 3 years of MDGs implementation; the explanation could be that the later target (5%) was set using data issued by surveys conducted in sentinel sites with a high exposure risk.

***Target 8: By 2015, to have halted and begun to reverse the incidence of malaria and other major diseases***

**Indicator 21: prevalence of death rates associated with malaria**

Between 1995 and 2003, uncomplicated malaria represented over 50% of all consultations, whilst the mortality rate for malaria cases was 51% in 2000 [2]. In 2012, malaria cases were only 5.2% and the mortality rate was 5% corresponding to 90% of relative reduction for morbidity and mortality [18]. This score reveals the fastened achievement of revised target set at 5% for Vision 2020 reflecting an impressive progress in halting and reversing the epidemic. Unfortunately, the disease is resurfacing since 2013/2014.

**Indicator 23: incidence associated with tuberculosis**

According to data on Tuberculosis (TB) for Rwanda in 2012/2013 [7], the indicator declined from 98/100,000 in 2000 to 69/100,000 in 2013; therefore, the incidence is halted and reversed.

***GOAL 7: ensure environmental sustainability***

***Target 10: by 2015, halve the proportion of people without sustainable access to safe drinking water***

**Indicator 29: proportion of population with sustainable access to an improved water source**

The indicator increased from 64.1% in 2000 to 84.8% in 2014 according to EICV4 2013/2014 surpassing the target set at 82% by 2015; the indicator has been revised upwards 100% by 2017/2018 [7].

***Target 11: by 2020, achieve a significant improvement in the lives of slum dwellers***

**Indicator 30: proportion of population with access to improved sanitation facilities:** The indicator increased from 51.5% in 2000 to 83.4% in 2014/2015 according to EICV4 2013/2014; thus, the basic target set by 2020 at 74.5% was achieved [12]. The Figure 4 where 2000 year's levels represent 100% illustrates the performance of main Rwanda MDGs indicators between 1990 and 2014/2015.

**Contributing factors:** The literature review shows that amongst numerous contributing factors of the above achievements, the good governance, especially good governance in health is unanimously ranked first. This is in accordance with the study on the good governance and health [19]. The literature review shows also that Vision 2020 [5] is one of potential contributing factors. The implementation of derived Health Vision 2020 aiming at providing sustainable financial resources for universal access to health care for all citizens" i.e. Universal Health Coverage (UHC) by 2020 stimulated positive health-related MDGs outcomes. Derived health sector reform [20], sound health policies and strategies lead to UHC implementation and stimulated improvement of health-related MDGs performance. Therefore, the Vision 2020 is also unanimously deemed potential contributing factor. The sections below examine closely the role played by Goal 8 and UHC implementation as third and fourth contributing factors respectively, assuming that the aforementioned respective roles of good governance and Vision 2020 are indubitable.

**GOAL 8: implementation as third contributing factor**

Our results on Goal 8 are not effective measures focusing only on target 13 as originally formulated i.e. *"Make essential drugs available and affordable to all who need them"*. Rather, our results intend to examine the role played by Goal 8 on “Global Partnership for Development” in the Rwanda health sector priorities, and on its comparison with WHO African Region and with WHO Global. According to World Health Statistics 2015 Part II, the total health expenditure per inhabitant per year was US$9 for Rwanda in 2000, US$34 for WHO African Region and US$487 globally. The external resources for health as share (%) of total health expenditure on health was estimated at 52% in 2000 for Rwanda, at 7% for the WHO African Region and at 0.3% globally [21]. For Rwanda, this total per capita allocation was too low compared to US$34 constant prices in 1997 as recommended by the 2000 WHO's Commission on Macroeconomics and Health. According to the Rwanda National Health Accounts 2006, this target was almost attained by Rwanda in 2006 with US$33.9 (100%) out of which households contributed US$ 8.8 (25.96%), the private sector US$0.7 (2.06%), Donors US$18(53.10%) and the Government of Rwanda (GOR) US$6.4 (18.88%). According to Global Health Statistics 2015- Part II [22], the total health expenditure per capita for Rwanda was US$ 70 in 2012 out of which 45.1% was funded by external resources versus 52% of US$9 in 2000. The 2012 amount is two times higher than the 2000 WHO's Commission Target worth US$34 and near eight times higher than the 2000 Rwanda level worth US$9. For the WHO African Region, the total health expenditure per capita ratio was estimated at US$105 in 2012 out of which 11.5% was funded by external versus 7% of US$34 in 2000. For Global, 0.3% out of US$487 was registered in 2000 versus 0.5% out of US$1025 in 2012. Therefore, relatively, for the periods 2000-2012, Rwanda benefited from external funding more than globally while nominally it is less funded than the both WHO regions. According to the Mid Term Review 2011 of HSSP II [23] from 2006 to 2011, 29% and 37% out of all external assistance went to Performance-Based Financing (PBF) and to Community Based Health Insurance (CBHI) respectively, demonstrating the importance of these two health reform drivers on which Rwanda UHC is built, with regards to external funding allocation during the MDGs 2015 lifetime. Thanks to the efforts of the GOR and the International Community partnering in health, the total health budget as share of total national budget (%) increased from 8.5% in 2000 to 16.05% in 2012/2013 and to 24% in 2013/2014 [21, 24] surpassing the 15% required under Abuja Declaration 2001.This share increased from 7.9% to 11.4% and from 12.9% to 14.1% in 2000 and 2012 for WHO African Region and WHO Global respectively. The required resources availability, from internal and via Goal 8 implementation, their efficient management have gradually allowed Rwanda to fasten the attainment of current impressive health-related MDGs outcomes [25-27].

**UHC implementation as fourth contributing factor:** The WHO defines UHC as ensuring that (i) all people can use the promotive, preventive, curative, rehabilitative and palliative (ii) that is defined as health services they need, of sufficient quality to be effective, (iii) while also ensuring that the use of these services does not expose the user to financial hardship [28]. Precisely, the three dimensions of UHC as presented above are described in the WHO conceptual framework on the WHO “UHC Cube”. This framework describes: (i) who is covered; (ii) which services are covered; and (iii) which proportion of the costs is covered (financial-risk protection) [29] The literature review shows that, thanks to the MDGs 2015, the Rwanda UHC was implemented before the adoption of the World Health Assembly (WHA) 58/2005/REC/1 issued by the Fifty-eighth WHA held in Geneva from the 16th to the 25^th^ of May 2005. This was steered by moving away from user fees towards sustainable health financing and universal coverage using social health insurance. The GOR anticipated the UHC implementation through health reform, integrating health financing and health service delivery, quality health care and equity [7], namely: (i) Community-Based Health Insurance (i) Community-Based Health Insurance (CBHI) [30-32] (ii) Performance-Based Financing (PBF) [33-35] now linked with accreditation; the built [23, 26, 27]; (iii) Quality Assurance of health services (QA) [7, 36] and (iv) the Community Health Program [37-40] The gradual implementation of health financing mechanisms and health interventions people-centered deriving from Health Vision 2020 allowed R wanda to better performing towards current Rwanda UHC level while stimulating the health-related MDGs outcomes [7, 27, 28, 41].

## Discussion

The discussion of results is mainly framed around the comparison of the Rwanda current status with the WHO African Region as, on average, one of lower performing standards amongst developing countries; and with the WHO Global deemed, on average, the higher performing standard. As Rwandan health-related indicators were ranked amongst the worst in 1990, situation exacerbated by the 1990s horrors, the achievement of respective basic or revised own target is considered as good performance; the attainment of the global level or the fastened attainment of the 2020 targets is considered as remarkable performance while the persisting gap, if any, is considered as weakness.

**Indicator 1: proportion of population living below the poverty line:** The official indicator allowing comparison of poverty level worldwide is the Population living on less than a $1 Power Purchasing Party (PPP) international dollar a day (<$ 1PPP int. $). For the 2007-2013 period, the indicator was 63% for Rwanda, 47% for African Region, 43.6% for Low Income Countries, 22.7% for Lower Middle Income Countries (LMIC) and 14.6% for the Global level, showing that Rwandan poverty line is higher than all WHO regions [38] and thus, the weakness is here considered.

**Indicator 4: prevalence of underweight children under-five years of age:** Registers 9% in Rwanda in 2014/2015 [14] surpassing the basic target set at 14.5% ,and thus the remarkable performance is noted. In sub-Saharan Africa, the underweight rate has fallen from 29% in 1990 to 20% in 2015, i.e. only 31.03% instead of 50% required. Worldwide, the rate decreased from 25% in 1990 to 14% in 2015, i.e. 44% still less than 50% required [4].

**Height-for-age (stunting):** Currently, thirty eight percent (38%) of Rwandan children under-five years of age are stunted. The indicator decreased from 48% in 1990 to 38% in 20114/2015 i.e. reduced by 21%; the basic target set at 24%, i.e. the half of the 1990 level is not achieved, and thus the weakness is noted. According to UN MDGs Report 2015, for all WHO regions, the indicator decreased except in WHO African Region where the number increased by about one third between 1990 and 2013 [4]; the related exact data is not retrieved for the two WHO regions.

**Weight-for-height (wasting):** For Rwanda, the indicator is 2% by 2014/2015 [14]; the target set exactly at 2% by 2015 is achieved. For the periods 2007-2014, Rwanda registered 3%, 10.3% for SSA and 7.7% for Global [4]. This nutritional condition is seen as remarkable performance.

**Indicator 13: under-five mortality rate per 1000 live births:** The Rwandan indicator estimated at 50/1,000 in 2014/2015 [14] is between the regional worth 90.1/1000 and the global worth 45.6 deaths/1000 [22]. Having achieved exactly the basic target set at 2015, it is to be considered as good performance.

**Indicator 14: infant mortality rate per 1000 live births:** The indicator was estimated at 32 deaths/1000 in 2014/2015 (RDHSV) from 85/1000 in 1990, 137/1000 in 1994 and 107/1000 in 2000. Even though the basic revised and revised targets set at 28 and 25 deaths/1000 by 2015 respectively are not achieved, the indicator has reached and surpassed a bit the 33.69/1000 live births reported for the global level [22] and thus, considered as remarkable performance.

**Indicator 15: proportion of one-year-old children immunized against measles:** The indicator was estimated at 95.2% in 2014/2015 [14] versus 95% as revised target and thus achieved. The comparison with WHO African Region and globally shows that 74% and 84% among one-year-olds were immunized against measles in 2013 respectively [22], exhibiting remarkable performance.

**Indicator 16: MMR per 100,000 live births:** For Rwanda, the indicator was estimated at 210/100,000 in 2014/2015 [14] versus 268 /100,000 as basic target and qualified as remarkable performance. In effect, the indicator has reached exactly the global level i.e. 210 deaths /100,000 live births whilst the WHO African Region accounted 960 deaths/100,000 [22].

**Indicator 17: proportion of births attended by skilled health personnel:** For Rwanda, the indicator was 90.7% in 2014/2015 [14] versus its own basic target set at 75% and thus considered as remarkable performance. In effect, the Rwandan indicator surpasses the WHO African Region's worth 51% between 2007 and 2013 [22] and the global one worth 74% in 2014 [4].

**HIV prevalence amongst men and women aged 15-49 years:** With regards to HIV prevalence, the indicator declined from 2,554/100,000 to 1,659/100,000 for Rwanda, from 3,221/100,000 to 2,669/100,000 for WHO African Region, and increased from 490/100,000 to 500/100,000 for WHO Global, between 2001 and 2013 [22].The Rwanda HIV/AIDS prevalence is better than WHO African Region and worse than globally. Besides, the fastened achievement of 3% since 2005 versus the target set at 5% by 2020 is found as remarkable performance.

**Indicator 21: prevalence of death rates associated with malaria:** For Rwanda, the malaria morbidity and mortality reduced by 90% from 2000 to 2012. Between 2000 and 2015, the global malaria incidence rate has fallen by an estimated 37% and the mortality rate by 58% [22]. The malaria incidence accounted 5,673/100,000 for Rwanda, 18,526/100,000 for the WHO African Region i.e. three times higher than Rwanda, and 3,744/100,000 worldwide in 2013/2014 i.e. to-third lower than Rwanda [22]. Rwanda is less affected by the epidemic than WHO African Region and more affected than Global. The fastened attainment of the target set at malaria morbidity worth 5% by 2020 versus 50% between 1995-2003 is considered as remarkable performance.

**Indicator 23: incidence, associated with tuberculosis:** The TB incidence rate per 100,000 declined by 30% from 98/100,000 in 2000 to 69/100,000 in 2013 in Rwanda while it declined from 337/100,000 to 280/100,000 in 2013 for the WHO African Region and from 152/100,000 in 2000 [22] to 126/100,000 worldwide. The two WHO regions are more affected by the TB than Rwanda and thus, the remarkable performance is noted.

**Indicator 29: proportion of population with sustainable access to an improved water source:** The indicator was estimated at 84.8% in 2014 ( EICV4) [12] versus the target set at 82% by 2015 and thus, the good performance is here noted; the indicator was estimated at 66% and at 91% in 2014 for WHO African Region and for WHO Global respectively, Rwanda is more served than WHO African Region and is less served than globally.

**Indicator 30: proportion of population with access to improved sanitation facilities:** The indicator increased from 51.5% in 2000 to 83.4% in 2014/2015, surpassing the WHO Global which increased from 54% to 68% between 1990 and 2015; Rwandan higher sanitation conditions than Global is to be considered as a remarkable performance. Following indicators are not discussed above: (i) Neonatal Mortality indicator worth 20 deaths/1000 versus 44.7 deaths/1000 and 30.5 deaths/1000 for WHO African Region and WHO Global respectively, the remarkable performance is noted; (ii) Antenatal care (4ANC) indicator worth 43.9% versus related target set at 50% by 2015 and (iii) contraceptive prevalence (modern methods) indicator worth 54% versus revised target set at 70% by 2015 [7] are considered as 'weaknesses'.

## Conclusion

The performance of Rwanda on the health-related Millennium Development Goals (MDGs) 2015 targets is impressive despite the negative effects of the 1990-1994 Civil War and the 1994 genocide against the Tutsi on basic 1990's levels. In effect, out of 17 health-related MDGs indicators and sub-indicators assessed including the three not discussed, eleven attained global levels or fastened attainment of the 2020 targets, two achieved basic or revised country targets and only four indicators show persisting gap. The good governance in health, the health Vision 2020 effective implementation, consistent mobilized resources from internal and external, and Rwanda Universal Health Coverage implementation have greatly contributed and stimulated improvement of above impressive health-related MDGs performances. Gaps still observed should be prioritized while implementing the emerging Sustainable Development Goals and the current gains should be preserved and improved.

### What is known about this topic

- The status of some health-related MDGs isolated targets in Rwanda.

### What this study adds

- Presentation of the status of health-related MDGs targets in Rwanda in a more holistic manner;
- Comparison of the status of health-related MDGs targets in Rwanda with regional and global levels;
- Discussion on possible contributing factors of Rwandan performance in achievement of health-related MDGs targets.

## Competing interests

The authors declare no competing interests.

## Authors’ contributions

Médard Nyandekwe had the initial idea for the study and he collected data. Médard Nyandekwe, Jean Baptiste Kakoma and Manasse Nzayirambaho contributed to the design of the study, data analysis, writing and review of the manuscript. All authors read the draft, provided feedback, and approved the final draft.
